# Genital and urinary tract malformations, associated morbidity, and need for urogenital surgery among children with esophageal atresia: a retrospective cohort study

**DOI:** 10.1007/s00383-026-06403-9

**Published:** 2026-04-09

**Authors:** Emma Svensson, Freja Eriksson, Lars Hagander, Erik Omling, Mark Bremholm Ellebæk, Martin Salö

**Affiliations:** 1 Pediatrics, Department of Clinical Sciences Lund, Lund University, Lund, Sweden; 2 Pediatric Surgical Sciences, Department of Women’s and Children’s Health, Uppsala University, Uppsala, Sweden; 3 Department of Pediatric and Adolescent Surgery, Skåne University Hospital, Lund, Sweden; 4 Centre of Excellence in Gastrointestinal Diseases and Malformations in Infancy and Childhood (GAIN), Odense University Hospital, University of Southern Denmark, Odense, Denmark; 5 Research Unit for Surgery, Department of Clinical Research, University of Southern Denmark, Odense, Denmark

**Keywords:** Esophageal atresia, Genital malformation, Urinary tract malformation, VACTERL, CAKUT, Kidney failure

## Abstract

**Purpose:**

Genital malformations are increasingly recognized when a VACTERL is present, but remain insufficiently described in children with esophageal atresia (EA). This study aimed to determine the prevalence and spectrum of genital and urinary tract malformations in children with EA, and to describe associated morbidity, need for additional surgery, and association with Gross classification.

**Methods:**

Retrospective cohort study including all children undergoing surgery for EA at a tertiary center of pediatric surgery between 2012–2023. Children deceased within five days of birth were excluded. Data were collected from medical records and analyzed using Chi^2^ and Fisher’s exact tests.

**Results:**

Ninety-three children were included. Genital and urinary tract malformations occurred in 12 (12.9%) and 18 (19.4%) children. In children with a VACTERL association, the prevalence was 29% (9/31) and 51.6% (16/31), respectively. Urinary tract infections, neurogenic bladder, and intermittent catheterization were more common in children with a genital or urinary tract malformation. Additional urogenital surgery was required in 50% of affected children. There was no significant association with Gross classification.

**Conclusion:**

Genital and urinary tract malformations are common in EA, contributing to morbidity and need for additional surgery. Screening for genital malformations should be considered, especially when a VACTERL association is present.

## Introduction

Esophageal atresia (EA) is the most common malformation of the esophagus, with a birth prevalence of approximately 1:4000 [1–3]. Advances in neonatal care and surgical management have markedly improved survival, shifting clinical focus towards associated anomalies and long-term morbidity [4]. Half of children with EA have one or more additional malformation and approximately a third present with a VACTERL association [3, 5]; a non-random cluster of malformations commonly defined as clinical or radiological findings consistent with three or more of the component features (vertebral anomalies, anorectal malformations, cardiac anomalies, tracheoesophageal fistula with esophageal atresia, renal malformations, and/or limb abnormalities) [6–8].

Renal, or urinary tract, malformations affect the kidneys, ureters, bladder, and/or urethra. These malformations are recognized in EA, and screening is common in clinical practice [9]. In contrast, genital malformations have received less attention, particularly among children who do not fulfill the criteria for a VACTERL association. While evidence suggests these malformations are more prevalent in VACTERL cases than previously recognized – especially in conjunction with anorectal or renal anomalies – data specifically focused on EA remains scarce[10–13]. To date, the few published studies on unselected EA cohorts furthermore report widely disparate prevalence rates of genital malformations of between 5.6 and 23.3% [12, 14, 15].

The clinical implications of genital malformations also vary widely. Although life-threatening disease is rare, they may contribute considerably to disease burden throughout life in affected individuals. In females, malformations can cause fertility issues, pain, pelvic outlet obstruction, spontaneous abortions, and preterm labor [16–19], while males may experience delayed puberty and increased risk for diabetes mellitus, cardiovascular disease, fertility issues, and testicular cancer [20, 21]. Additionally, a recent study showed that patients with genital malformations had substantially higher odds of developing neurogenic bladder than patients with tethered spinal cord, anorectal malformations, and renal anomalies [22]. Neurogenic bladder is, along with structural urinary tract malformations, the most common cause of kidney failure in children, and together they account for more than 50% of chronic and end-stage kidney disease in this patient group [23–25]. However, early diagnosis and proper management of malformations and their associated complications, such as neurogenic bladder, can decrease or even prevent future morbidity.

Despite recognition of the association between EA and urinary tract malformations, prevalence rates vary largely and anomalies are reported to occur in between 15 and 50% [3, 14, 26–28]. Furthermore, data describing the spectrum of genital malformations in unselected EA cohorts remain limited. Most importantly, the clinical implications and early morbidity associated with concomitant genital or urinary tract malformations in this patient group have not been assessed. The present study aims to address these gaps to refine current understanding, and to inform risk stratification and follow-up strategies in children with EA. The specific aims were therefore to (1) describe the prevalence and spectrum of genital and urinary tract malformations in children with EA, with and without a VACTERL association; (2) characterize early urinary tract-related morbidity and need for urogenital surgery in children with EA, stratified by the occurrence of genital or urinary tract malformations; and (3) explore potential associations between genital and urinary tract malformations and EA subtype according to the Gross classification.

## Materials and methods

### Study design and setting

This was a retrospective single-center cohort study of children undergoing surgery for EA between 2012 and 2023. The study was performed in accordance with the Strengthening Reporting of Observational Studies in Epidemiology (STROBE) guidelines [29].

The study was conducted at the Department of Pediatric and Adolescent Surgery at Skåne University Hospital in Lund, Sweden; a tertiary care center and, since 2018, one of two referral hospitals for children with EA in Sweden. According to local institutional protocol, children with EA referred to the department are routinely evaluated for VACTERL component features and other associated anomalies. The screening program includes a preoperative echocardiography, an intraoperative tracheoscopy (implemented in the screening program in 2018), an ultrasound of the kidneys, urinary tract and spinal canal (performed pre- or postoperatively), and a conventional radiography of the vertebral column and sacrum[30]. Additional diagnostic investigations are performed if indicated. No standardized assessment for genital malformations is currently implemented, apart from the routine clinical examination to screen for undescended testis in boys.

### Study participants

All children undergoing surgery for EA between 2012 and 2023 at Skåne University Hospital were eligible for inclusion. Children were identified through the digital surgery planning system (Orbit^Ⓡ^), and social security numbers were obtained. Children who died within five days of birth were excluded, as evaluation for associated anomalies was not feasible. All study participants were followed until death or end of the study (February 2024).

### Data collection

Data were extracted from the regional electronic medical record system (MeliorⓇ). For children residing outside the Skåne region, data were additionally retrieved through the Swedish National Patient Overview system. Predefined variables were collected through systematic review of imaging reports, operative notes, outpatient and inpatient records, laboratory data, and ICD-10 diagnostic codes.

### Primary and secondary outcomes

The primary outcome was the prevalence and type of an associated genital or urinary tract malformation. Secondary outcomes comprised urinary tract-related morbidity (urinary tract infections (UTIs), neurogenic bladder, enuresis, use of anticholinergic drugs, need for intermittent catheterization, and impaired kidney function) and urogenital surgery.

### Additional variables

Demographic variables included sex, year of birth, age at follow-up, gestational age at birth, EA subtype according to the Gross classification[31], and presence of additional anomalies (vertebral, anorectal, cardiac, and limb anomalies, or tethered spinal cord).

### Definitions

A genital malformation was defined as any minor or major malformation to the internal or external genitalia, including undescended testis. A urinary tract malformation was defined as any minor or major malformation affecting the kidneys, ureters, bladder, or urethra. Distal urethral anomalies such as hypospadias were classified as genital malformations.

A UTI included acute cystitis and pyelonephritis, and was defined by a positive urine culture, medical record entries, or ICD-10 codes. The number of UTIs and need for prophylactic antibiotic treatment was noted. Neurogenic bladder and enuresis were defined based on clinical documentation or ICD-10 codes. Use of anticholinergic medication was defined as in- or outpatient prescription for lower urinary tract symptoms. Intermittent catheterization included short- and long-term catheterization for bladder management. Impaired kidney function was defined as either receiving an ICD-10 code of chronic or end-stage kidney disease or being referred to the Department of Pediatric Nephrology for decreased kidney function. A urogenital surgical procedure was defined as any surgical intervention involving the genital organs or urinary tract performed under general anesthesia.

A VACTERL association was defined as EA plus at least two additional component features. Both major and minor malformations within each organ system were considered a VACTERL component, in accordance with the definition proposed by van de Putte et al. [32]. Tethered spinal cord was analyzed separately. Malformations were considered absent if not documented in medical records or ICD-10 coding.

### Statistical analysis

Descriptive statistics are presented as counts and percentages or medians with total range. Associations between genital and urinary tract malformations and concurrent anomalies, urinary tract-related morbidity, urogenital procedures, and Gross classification were analyzed using a Chi^2^ test. For groups with five or fewer individuals, a Fisher’s exact test was used. In cases of missing data, the individual was excluded from the specific analysis. A two-sided p-value of < 0.05 was initially considered statistically significant. To account for multiple subgroup comparisons and minimize the risk of Type I errors, a Bonferroni correction was applied. Consequently, the significance threshold was adjusted to a p-value of 0.0015. Statistical analyses were performed using SPSS version 29 (IBM Corp., Armonk, NY, USA).

### Ethical declaration

The study was conducted in accordance with the Declaration of Helsinki and approved by the Regional Ethical Review board in Lund (2010/49). Permission to access medical records and utilize patient data was granted by the Skåne University Hospital’s Committee for Quality Registers and Health Care Databases (KVB; 2019/19).

## Results

### Patient characteristics

A total of 95 children underwent surgery for EA during the specified period. Two died within five days of birth due to cardiac anomalies and were excluded from further analysis. The final cohort included 93 children, of which 61.3% (57/93) were boys. The median follow-up time was five years (total range 0–12 years). A total of 86% (80/93) had a Gross type C and 33.3% (31/93) fulfilled the conditions for a VACTERL association. Three children (3/93; 3.2%) died after inclusion, all before the age of five months and all with at least one concomitant malformation. Patient characteristics are presented in Table 1.

**Table 1:** Demographics, Gross classification, and related malformations among 93 children undergoing surgery for esophageal atresia between 2012 and 2023

Variables	Number of cases (%)
Sex	
Girls	36 (38.7)
Boys	57 (61.3)
Age at follow-up (years)	
≤ 5	49 (52.7)
6–10	40 (43)
> 10	4 (4.3)
Age at follow-up (years) – median (range)	5 (0–12)
Deceased	3 (3.2)
Gestational week < 37^1^	24 (25.8)
Gross classification^2^	
A	4 (4.3)
B	1 (1.1)
C	80 (86)
D	1 (1.1)
E	4 (4.3)
Esophageal atresia without other malformations	37 (39.7)
VACTERL association	31 (33.3)
Vertebral anomaly	30 (32.3)
Anorectal malformation	14 (15.1)
Cardiac anomaly	31 (33.3)
Urinary tract malformation	18 (19.4)
Limb abnormality	9 (9.7)
Tethered spinal cord	12 (12.9)

Values presented as the absolute number and percentage of total cohort (93) in parenthesis unless indicated otherwise

^1^Data missing for one child

^2^Data missing for three children

### Genital malformations

Genital malformations were seen in 12 children (12/93; 12.9%); four girls and eight boys (4/36; 11.1% and 8/57; 14%, respectively). In children with a VACTERL association, the prevalence was 29% (9/31). Among girls, two had double vagina, and two had a combination of vaginal agenesis and fused labia. Of the eight boys affected, five (62.5%) had undescended testis and two (25%) had hypospadias; one had a combination of the two conditions. Of the six children with undescended testis, all but one were born term. A description of identified anomalies is presented in Table 2.

**Table 2:** Spectrum of genital and urinary tract malformations identified in 93 children undergoing surgery for esophageal atresia between 2012 and 2023

Type of malformation	Number of cases (%)
**Genital malformations**	12 (12.9)
Girls^1^	4 (11.1)
Double vagina	2 (5.6)
Vaginal agenesis	2 (5.6)
Fused labia	2 (5.6)
Boys^2^	8 (14)
Undescended testis	6 (10.5)
Hypospadias	3 (5.3)
**Urinary tract malformations**	18 (19.4)
Renal fusion anomalies	6 (6.3)
Vesicourethral reflux	6 (6.3)
Single kidney	4 (4.3)
Duplex anomalies	3 (3.1)
Ectopic kidney	2 (2.1)
Multicystic dysplastic kidney	2 (2.1)
Ureteropelvic junction obstruction	1 (1.1)
Renal rotational anomalies	1 (1.1)

^1^Absolute number and percentage of the total cohort of girls (36). ^2^Absolute number and percentage of the total cohort of boys (57). Malformations described are not mutually exclusive

Children with a genital malformation significantly more often had anorectal malformations (58.3% versus 8.6%, p < 0.001) and tethered spinal cord (50% versus 7.4%, p < 0.001), and were more likely to have a VACTERL association (75% versus 27.2%, p < 0.01) or a vertebral anomaly (66.7% versus 27.2%, p < 0.01), when compared to children without a genital malformation. Eight out of twelve (66.7%) with a genital malformation had a concurrent urinary tract malformation, compared to ten (12.3%) among children without a genital malformation (p < 0.001). Results on genital malformations are detailed in Table 3.

**Table 3:** Characteristics and related malformations among 93 children undergoing surgery for esophageal atresia between 2012 and 2023, with and without an identified genital or urinary tract malformation

	Genital malformation			Urinary tract malformation		
	Present (n = 12)	Not present (n = 81)	p-value	Present (n = 18)	Not present (n = 75)	p-value
Sex			0.76			0.58
Girls	4 (33.3)	32 (39.5)		8 (44.4)	28 (37.3)	
Boys	8 (66.7)	49 (60.5)		10 (55.6)	47 (62.7)	
Age at follow-up (years)			0.61			0.61
≤ 5	5 (41.6)	44 (54.3)		10 (55.6)	39 (52)	
6–10	7 (58.3)	33 (40.7)		8 (44.4)	32 (42.7)	
> 10	0 (0)	4 (4.9)		0 (0.0)	4 (5.3)	
Prenatal diagnosis	0 (0)	-	-	6 (33.3)	-	-
Gestational week < 37^1^	5 (41.7)	19 (23.8)	0.86	5 (27.8)	19 (25.3)	0.86
Gross classification^2^			0.86			0.49
A	1 (8.3)	3 (3.8)		2 (11.1)	2 (2.7)	
B	0 (0)	1 (1.3)		0 (0)	1 (1.3)	
C	11 (91.7)	69 (88.5)		14 (77.8)	66 (88)	
D	0 (0)	1 (1.3)		0 (0)	1 (1.3)	
E	0 (0)	4 (5.1)		1 (5.6)	3 (4)	
VACTERL association	9 (75)	22 (27.2)	< 0.01	16 (88.9)	15 (20)	< *0.001*
Vertebral anomaly	8 (66.7)	22 (27.2)	< 0.01	11 (61.1)	19 (25.3)	< 0.01
Anorectal malformation	7 (58.3)	7 (8.6)	< *0.001*	9 (50)	5 (6.7)	< *0.001*
Cardiac anomaly	6 (50)	25 (30.9)	0.21	8 (44.4)	23 (30.7)	0.27
Urinary tract malformation	8 (66.7)	10 (12.3)	< *0.001*	–	–	–
Limb abnormality	2 (16.7)	7 (8.6)	0.33	2 (11.1)	7 (9.3)	0.82
Tethered spinal cord	6 (50)	6 (7.4)	< *0.001*	7 (38.9)	5 (6.7)	< *0.001*
Genital malformation	–	–	–	8 (44.4)	4 (5.3)	< *0.001*

*p*-values meeting the signifi cance level of < 0.0015 are italized

### Urinary tract malformations

A urinary tract malformation was found in 18 children (18/93; 19.4%). Among children with a VACTERL association, the prevalence was 51.6% (16/31). The most common malformations were vesicourethral reflux and renal fusion anomalies, with six affected children (6/18; 33.3%) respectively. Other malformations included single kidney, duplex anomalies, ectopic kidney, multicystic dysplastic kidney, ureteropelvic junction obstruction, and rotational anomalies (Table 2). Four children (4/93; 4.3%) had multiple urinary tract malformations.

There was no significant difference in sex (males 55.6% and 62.7%, p = 0.58) or prematurity (27.8% and 25.3%, p = 0.86) between children with and without a urinary tract malformation. Compared to children without a urinary tract malformation, children with a urinary tract malformation were more likely to have a VACTERL association (88.9% versus 20%, p < 0.001), anorectal malformation (50% versus 6.7%, p < 0.001), tethered spinal cord (38.9% versus 6.7%, p < 0.001), or a genital malformation (44.4% versus 5.3%, p < 0.001). Results on urinary tract malformations are detailed in Tables 2 and 3.

Chi^2^ test and Fisher’s exact tests. Values presented as absolute numbers and percentages in parenthesis. ^1^Data missing for one child. ^2^Data missing for three children.

### Urinary tract-related morbidity

Children with genital or urinary tract malformations had a higher frequency of UTIs compared to children without these malformations (41.2% versus 8.7%, p < 0.01 and 50% versus 4%, p < 0.001, respectively). Recurrent UTIs occurred in six children (6/93; 6.5%), all of whom had a urinary tract malformation. Three required prophylactic antibiotic treatment with trimethoprim-sulfamethoxazole. Neurogenic bladder was more common among children with genital or urinary tract malformations (25% versus 3.7%, p = 0.03 and 22.2% versus 2.7%, p = 0.01, respectively), and need for intermittent catheterization was observed more frequently in these groups (16.7% versus 1.2%, p = 0.04 and 16.7% versus 0%, p < 0.01, respectively). One child with a urinary tract malformation was referred to the Department of Pediatric Nephrology due to impaired kidney function. Results on urinary tract-related morbidity are detailed in Table 4.

**Table 4:** Urinary tract-related morbidity among 93 children undergoing surgery for esophageal atresia between 2012 and 2023, with and without an identified genital or urinary tract malformation

	Genital malformation			Urinary tract malformation		
	Present (n = 12)	Not present (n = 81)	p-value	Present (n = 18)	Not present (n = 75)	p-value
Urinary tract infection	5 (41.2)	7 (8.7)	< 0.01	9 (50)	3 (4)	< *0.001*
Neurogenic bladder	3 (25)	3 (3.7)	0.03	4 (22.2)	2 (2.7)	0.01
Anticholinergic medication	1 (8.3)	1 (1.2)	0.24	1 (5.6)	1 (1.3)	0.35
Intermittent catheterization	2 (16.7)	1 (1.2)	0.04	3 (16.7)	0 (0)	< 0.01
Enuresis	2 (16.7)	3 (3.7)	0.12	3 (16.7)	2 (2.7)	0.05

*p*-values meeting the signifi cance level of < 0.0015 are italized

Fisher’s exact test. Values presented as absolute numbers and percentages in parentheses

Six children with genital malformations (6/12; 50%) and seven with urinary tract malformations (7/18; 38.9%) required urogenital surgery during the study period, whereas no such procedures were recorded among children without an identified genital or urinary tract malformation (p < 0.001 for both comparisons). The type and frequency of surgical interventions are described in Table 5.

**Table 5:** Type and frequency of urogenital surgical interventions in 93 children undergoing surgery for esophageal atresia between 2012 and 2023

Surgical interventions	Number of cases (%)
**Genital surgery**	6 (6.5)
Girls^1^	2 (5.5)
Vaginal reconstruction	1 (2.8)
Vaginal septal resection	1 (2.8)
Boys^2^	4 (7)
Orchidopexy	3 (5.3)
Hypospadias repair	1 (1.8)
**Urinary tract surgery**	7 (7.5)
Suprapubic catheter insertion	5 (5.4)
Nephroureterectomy	2 (2.2)
Dilation of urethra	1 (1.1)
Ureteroneocystostomy	1 (1.1)
Deflux injection	1 (1.1)
Creation of neourethra	1 (1.1)
Ureteral stenting	1 (1.1)
Ureterocutaneostomy	1 (1.1)

^1^Absolute number and percentage of the total cohort of girls (36). ^2^Absolute number and percentage of the total cohort of boys (57). Interventions described are not mutually exclusive

### Association with type of esophageal atresia

There was no statistically significant association between the presence of a genital or urinary tract malformation and Gross classification (p = 0.86 and p = 0.49, respectively; Table 3).

## Discussion

In this study, we have described the frequency of genital and urinary tract malformations and associated early morbidity in all consecutive children undergoing repair for EA at a tertiary care center over a twelve-year period. Almost one in eight in this cohort had a genital malformation, and one in five had a concomitant urinary tract malformation. The prevalences of genital or urinary tract malformations were, as expected, higher among those with a VACTERL association, as it was seen in almost a third and more than half of the children. Genital and urinary tract malformations contributed to the overall morbidity, as 27.3% of affected children had recurrent UTIs during preschool age and early school age, 18.2% suffered from neurogenic bladder, and 13.6% required intermittent catheterization. In addition, 50% of children with a genital or urinary tract malformation required at least one additional surgical procedure during the study period. These findings suggest an increased early procedural and morbidity burden in this group, although causality cannot be inferred.

While the risk for concomitant urinary tract malformations is known, the prevalence of genital malformations differed from previous estimates [12, 14, 15]. To our knowledge, only three studies have assessed the occurrence of genital malformations in an unselected group of children with EA [12, 14, 15]. Different inclusion criteria and shorter follow-up time may explain some of the differences: Stoll et al. followed children until one and two years of age, respectively, and reported prevalences of 6.1% and 5.6%[14, 15]. This should be compared to 12.9% in our study, where children were followed for a median of five years, and minor anomalies such as undescended testis were included. However, we suspect that the number reported in the present study is still an underestimation of the true prevalence considering that genital malformations were not routinely screened for in our cohort, and that anomalies may not be evident until puberty, or even later in life. This interpretation is supported by a study published by de Jong et al., in which the follow-up period was up to 18 years, concluding a considerably higher prevalence of genital malformations at 23.3% [12].

Consistent with previous studies, our data support that genital malformations are common when there is a VACTERL association [12, 13, 33–35]. A prior study has reported genital malformations in between 24 and 38% of patients with EA as part of a VACTERL association depending on what subgroup is described, compared to our findings of 29%[34]. The variations in these numbers are again likely explained by different inclusion criteria and study populations; Hambraeus et al. assessed genital malformations in VACTERL patients with EA and/or an anorectal malformation, but did not present findings from an unmixed group of children with EA. Consistent with current evidence, genital malformations were associated with anorectal and renal malformations, but not cardiac or limb defects [11, 34].

Most urinary tract-related morbidities that were assessed in our cohort were more common among children with both genital and urinary tract malformations. These morbidities may not be solely attributable to genital or urinary tract malformations, as conditions such as tethered spinal cord, vertebral anomalies, and anorectal malformations – all more common in children with genital or urinary tract malformations in our cohort – are known risk factors for UTIs, neurogenic bladder, and intermittent catheterization [36–38]. Due to the nature of this study and the inherently limited sample size, a multivariable regression analysis to adjust for potential confounders was not feasible as the events-per-variable assumption of more than ten outcome events was not met, and independent associations could not be established. These findings should therefore be interpreted with caution. Nevertheless, having a genital or urinary tract malformation put children at risk for additional surgical procedures.

It is well-established that children with urinary tract malformations have an increased risk of developing chronic kidney disease, and that they develop this at a younger age than healthy controls [25]. Only one patient in our cohort developed impaired kidney function. While urinary tract malformations and neurogenic bladder are recognized risk factors for chronic kidney disease [39], the relatively short follow-up in this study precludes assessment of long-term renal outcomes, and the low prevalence of renal impairment likely reflects the young age of the cohort. Longitudinal follow-up into adolescence and adulthood utilizing population-based registers or prospectively collected data is necessary to determine the lifetime renal impact of these malformations.

Our findings emphasize the importance of considering associated malformations in children with EA. While urinary tract malformations are routinely assessed for in most referral centers, genital malformations may not be evident on initial examination or routine diagnostic imaging. In boys, clinical examination to detect undescended testes is widely recommended [40–42], and hypospadias will often be identified in a VACTERL screening. In girls, however, most genital malformations are internal and may require more specific clinical assessment or imaging, such as neonatal ultrasound, vaginoscopy, or diagnostic laparoscopy [33, 43]. It has been suggested that the used VACTERL acronym should be extended to include genital malformations [11, 13, 34], to recognize the potential disease burden from these conditions later in life [44]. While our study does not resolve this debate, it supports the notion that genital malformations represent a clinically relevant component of the phenotypic spectrum in EA, and consideration could be given to incorporating a structured assessment for genital anomalies into follow-up programs for children with EA, particularly in those with other VACTERL features. We suggest integrating an abdominal ultrasound of the internal genitalia into established renal and spinal neonatal ultrasound screening, alongside clinical evaluation of the outer genitalia and, if high clinical suspicion, a vaginoscopy [45]. For patients without initial anomalies, we propose follow-up at age ten, with continued vigilance for adolescent symptoms such as pelvic pain or other signs of obstructive disorders. However, these measures should be balanced against feasibility, resource implications, and the clinical significance of detected anomalies, and prospective studies are needed to define optimal screening strategies.

This study has several limitations. Its retrospective design entails risk of incomplete data capture, particularly for morbidities such as UTIs managed outside tertiary care. There is also potential ascertainment and surveillance bias, especially given evolving screening practices during the study period. Due to the national screening recommendation for children with EA, we believe most associated VACTERL components are recorded; however, genital malformations may still be underdiagnosed, and the true prevalence should be analyzed in prospective or population-based register studies. Such studies would additionally allow for a larger sample size and power, enabling multivariable analyses to thoroughly assess independent associations, as well as long-term clinical impact.

## Conclusion

This study confirms that genital malformations are common in children with EA when looked for, especially in those with a VACTERL association. Genital and urinary tract malformations contribute substantially to morbidity and need for additional surgery during childhood. Findings from this and recent studies provide evidence support that screening for genital malformations should be included as part of the VACTERL evaluation, to prevent morbidity related to delayed diagnosis, and to better inform patients and caregivers about the expected lifelong disease burden.

## Data Availability

Data supporting the results presented in this article will be made available by the corresponding author on reasonable request.
